# Cases of the Urticaria or Nettle Rash, with Observations

**Published:** 1794

**Authors:** T. M. Winterbottom

**Affiliations:** Physician to the Settlement at Sierra Leone.


VI. *Caſes of the Urticaria or Nettle Raſh, with Obſervations; by* T. M. Winterbottom, *M. D. Phyſician to the Settlement at Sierra Leone: communicated in a Letter to* Robert Willan, *M. D. Phyſician in London, and by him to Dr.* Simmons.

A Black woman, aged thirty-ſix years, ſtrong and healthy, after having eaten of a fruit which ſhe found in the woods, in form and ſize reſembling a damſon, and for which ſhe miſtook it, was next morning affected with a ſenſe of uneaſineſs and liſtleſſneſs over her whole body, accompanied with nauſea and oppreſſion at the præcordia; and with a ſenſation of creeping over the whole body, which was followed by a general tumefaction, eſpecially of the face. The

The nofe was remarkably fwoln, hot, itchy, and painful, particularly the alæ nafi; the upper lip was alfo much tumefied. She moreover complained much of a forenefs in the throat, attended with a troublefome tickling and fenfe of conftriction of the fauces, which rendered deglutition difficult. About the fame time an eruption took place over the whole body, more efpecially on the neck, breaft, and arms, of large red fpots elevated above the fkin, very hot, and intolerably itchy. On becoming warm in bed fhe felt as if ftung all over with nettles; and the fpots at that time appeared more numerous. The next morning fhe took a dofe of *fal. cathart. amar.* after the operation of which the tumefaction of the body was entirely removed, and the affection of the throat was very much relieved.

The eruption was much diminifhed in the courfe of the day, but did not entirely difappear until three or four days after, upon taking a fecond dofe of the falts. The itching continued troublefome for a week or two longer, and was always aggravated during the night.

Her hufband, who had eaten of the fame fruit, was fimilarly affected, except in feeling but but little forenefs of throat, and in having his face more fwoln, efpecially the lips.

A woman and child, whom I did not fee till after their recovery, were affected, according to their own report, in nearly the fame manner, from eating the fame kind of fruit; they recovered without any other medicine than a dofe of purging falts.

The tree which bears this fruit is, I believe, hitherto undefcribed; the fruit, when ripe, is not unlike a damfon, having a ftone within, being black, and of a fweetifh tafte. Its juice, which at firft is nearly colourlefs, gives a very durable black or dark-brown colour to linen.

An eruption fimilar to the above occurs from a variety of fubftances taken into the ftomach; it is commonly termed a furfeit, or by the vulgar in fome parts, poifoning. This may depend either upon an idiofyncracy with regard to certain articles of diet, as crabs, and fome other fhell fifh*; or it may arife from a mixture of different

* A man was always affected with urticaria after eating crabs, whether boiled or in foup; he was affected thus even by the vapour which arofe from them; as alfo by taking the lap. cancr. Dr. Tode, from whom this is quoted, (Med. chir.

different ſubſtances in the ſtomach, producing a noxious compound, though any of them ſingly would be harmleſs. Of this, Salmon, taken along with milk, affords an example.

Under the former head, perhaps nothing is more curious than the effects of the vegetable bitter. I have myſelf been twice violently affected by eating the ſweet Almond: the firſt time, within a few hours after eating this fruit, though in no great quantity, I was ſeized with ſlight nauſea, uneaſineſs in the ſtomach and bowels, without any fixed pain; great reſtleſſneſs and increaſed heat. Theſe ſymptoms were ſoon followed by an oedematous ſwelling of the face, eſpecially of the lips and noſe, which were extremely hot and itchy. There was, at the ſame time, an uneaſy tickling ſenſation in the throat, which excited a troubleſome cough and a conſtriction of the fauces, which ſeemed to threaten ſuffocation. The tongue likewiſe became enlarged and ſtiff, cauſing a ſlowneſs and faultering in the ſpeech. Soon after going to bed, an chir. bibl.) obſerves that he is himſelf frequently, though not always, affected with a ſlight urticaria after eating crab ſoup. — A woman by eating ſtrawberries was conſtantly affected with urticaria. See Vogel's Handbuch der Pract. Arzneywiſſenſchaft, vol. 3, p. 277.

eruperuption took place over the whole body, of ſpots nearly as large as a ſixpence, of a dead white colour, a little elevated above the ſkin like the wheals produced by the ſting of a nettle, and intolerably itchy. In their interſtices the ſkin was of a high red colour; the whole body was alſo tumefied, though in no great degree. Theſe ſymptoms continued during the greater part of the night, but gradually abated towards morning, upon the breaking out of a gentle perſpiration which was encouraged by warm diluents; the next day, not the leaſt veſtige of the complaint remained.

The ſecond time this affection occurred, the appearances were nearly the ſame, except that they began a few hours later than in the firſt inſtance — The eruption alſo continued the greater part of the enſuing day, and then gradually declined. From eating the blanched Almond I feel no inconvenience, but have never ventured ſince to eat them in their unblanched ſtate.

During my reſidence in Edinburgh, a patient was received into the clinical ward affected nearly in the manner above deſcribed; I ſhall tranſcribe his caſe from the clinical reports taken at the time.

" Nov. 19, 1787. Alex. Robertſon, aged 18,

" a Labourer; has a colourleſs puffy ſwelling over

" his whole body, but more eſpecially over his

" head, upper extremities and thighs. The

" ſwelling is pretty diſtinctly ſeen and felt upon

" the forehead, which pits upon preſſure ; the

" palpebræ and upper lip are likewiſe a little

" tumefied. The parts chiefly affected are fre-

" quently attacked with almoſt intolerable itchi-

" neſs, and when ſcratched have an appearance

" as if ſtung with nettles—pulſe natural; appe-

" tite good ; body regular. Two or three days

" previous to theſe complaints he was, at dif-

" ferent times, ſuddenly attacked with great

" vertigo, and on the 18th, firſt obſerved the

" ſwelling and itchineſs—He attributes his com-

" plaints to ſudden alternations of heat and cold.

" Has uſed no Medicines.

" 20. He took a doſe of Pulv. e. Jalap. co.

" which did not operate — on the 21ſt having

" had only one natural ſtool, the purgative was

" repeated. The ſwelling was nearly gone,

" but the itching continued—

" 22. Had three motions from the phyſic:—

" the itching had ceaſed on the head, and was eaſier

" on the trunk of the body ; but now began

" to affect the lower extremities—He was or-
" dered to employ the warm bath, and after-
" wards to take Pulv. Dover. gr. x with a dia-
" phoretic Julep occasionally.

" 23. He had sweated properly:—his voice
" and countenance were natural—the itching
" was gone from all parts but the legs and feet:
" the Julep was directed to be continued.

" 24. Some itching still remained about the
" legs and thighs; he was ordered to take next
" day a decoction of Tamarinds with Senna.

" 25. The itching was almost gone—

" On the 26th he left the house, cured."

Dr. Gregory, in his clinical lectures, mentioning this case, said he suspected it might have arisen from something disagreeable to the patient's stomach of the nature of the vegetable Bitter—The Doctor also observed that he had himself been subject to a similar complaint from eating Almonds; that he had a violent attack of fever, swelling of the body, and a copious eruption on the skin, attended with a loss of voice and coldness of the extremities, which, however, went off the next day — Nearly the same symptoms, he added, had occurred to him from eating a green cucumber with the skin upon it; and continued four days, but were at length removed by a cathartic.

About two years before this period, Doctor Gregory had feen, he faid, a patient affected in a fimilar way by drinking porter; he was feized with ficknefs, flight fever, head ach, and great itching over his body, occafioned probably by the bitter in the porter.—The patient Robertfon, whofe cafe is related above, alfo faid that he was taken ill foon after drinking fome porter.

The above difeafe appears to me to belong to the genus *urticaria*. Authors have divided urticaria into an acute and a chronic fpecies; the former has had various denominations, as *febris urticata*, *fcarlatina urticata*, *purpura urticata*, *febris rubra pruriginofa* — Some Writers, however, feem to have confined the term *urticaria* wholly to the chronic kind*—Thefe diftinctions appear unneceffary if we reflect how difficult it is to account for the effects of ftimuli upon different conftitutions; and how varioufly the difeafe may be modified, either by the peculiar irritability of the patient, or by a greater or lefs degree of activity in the irritating matter taken into the ftomach.—Befides, the term *febris urticata* is highly improper; for the fymptoms which

* See Medical Tranfactions, vol. II.

occur occur in the acute, or febrile ſtate, as it is called, are not thoſe of fever, but merely of erethiſmus, being the conſequences of a greater or leſs degree of irritation.

The *ſcarlatina, urticaria,* and *eſſera* have been conſidered, by ſome *, as diſeaſes of a ſimilar nature; but between ſcarlatina and the others, the difference is ſo ſtriking that it need not be minutely pointed out—The diſtinction between *urticaria* and *eſſera* does not appear ſo obvious—Some have conſidered *eſſera* only as a chronic ſtate of *urticaria,* but a much more eſſential diſtinction may be eſtabliſhed between the two diſeaſes—*Urticaria* ſeems always to be connected with an affection of the ſyſtem, and of the ſtomach and primæ viæ in particular, whereas the *eſſera* appears to be merely a local affection of the ſkin unconnected with any diſeaſe of the conſtitution.

Many perſons who have an irritable or delicate ſkin are liable, during the ſpring and ſummer ſeaſons, to frequent eruptions on the arms, face and neck, commonly called heat-ſpots, reſembling the ſtings of nettles, which obſerve no certain period of duration, but ſometimes

* See Selle Rudimenta Pyretologiæ.

quickly difappear, and fometimes are very permanent. A lady of my acquaintance is always affected with an eruption of this kind when her fkin is touched with the common wall flower. The bites of infects have confiderable effect in producing a fimilar eruption on the fkins of fome perfons, while others are little affected by them. Aftruc fays this *effera* is very common in Languedoc, but he confiders it as the fame with *urticaria*.

Thofe who account for the effects of the vegetable bitter, upon the fuppofition of its being carried into the blood before it exerts its action on the extreme veffels, afford little fatisfaction to pathological inquirers. How can we, indeed, imagine that fo fmall a portion of matter as is probably extracted from the fkins of a few almonds, fhould, when mixed with the mafs of circulating fluids, produce fuch powerful effects? It appears, therefore, more rational to conclude that the ftomach is the part primarily affected; and that the eruption is only a confequence of the clofe connexion which fubfifts between this organ and the fkin. That connexion is proved by a number of inftances; I fhall, however, adduce one more which is peculiar to the climate wherein I am at prefent fituated.

ſituated. This is the *prickly heat*, a papulous eruption which appears chiefly on parts covered by the clothes. Strangers are generally moſt liable to it, though ſome never have it, and yet enjoy good health. Fair people are more affected by it than thoſe of very dark complexions; yet I have ſometimes ſeen it affect even black people. This eruption is generally conſidered as a mark of health; from what I have ſeen of it, I am rather inclined to conſider it as indicating an active and healthy ſtate of the ſtomach. The ſlighteſt indiſpoſition or uneaſineſs of the ſtomach is commonly followed by a conſiderable abatement or total diſappearance of the eruption; in ſuch caſes, a doſe of bark, a glaſs of wine or any warm liquor, is frequently ſucceeded by a general tingling over the body, and a copious eruption of prickly heat; where the eruption is already out, it is often much increaſed and rendered more vivid by the ſame means.

It has often been ſuppoſed that bitters poſſeſs a narcotic quality; for which reaſon the gentian has been omitted in ſome of the foreign diſpenſatories. The long-continued uſe of bitters certainly tends to deſtroy the tone of the ſtomach and of the conſtitution; and thence produces irregularities in the circulation, and more eſpecially congeſtions in the head. The fatal effects aſcribed to the Portland powder may be thus accounted for; at leaſt with more probability than by the common hypotheſis of a repulſion of gouty matter from the extremities, and its conſequent depoſition upon other parts, of which we have no proper evidence.

*Free Town,*
*Sierra Leone,*
*June* 3, 1793.

